# Special Issue: Advanced Coatings for Corrosion Protection

**DOI:** 10.3390/ma13153401

**Published:** 2020-08-01

**Authors:** Wolfram Fürbeth

**Affiliations:** DECHEMA Research Institute, Theodor-Heuss-Allee 25, 60486 Frankfurt am Main, Germany; wolfram.fuerbeth@dechema.de; Tel.: +49-69-7564-398

**Keywords:** metallic coatings, anodizing layers, passivation, polymeric coatings, laser cladding, PVD, superhydrophobic coatings, composite coatings

## Abstract

Corrosion is an important issue in many industrial fields. Among others, coatings are by far the most important technology for corrosion protection of metallic surfaces. The special issue “Advanced Coatings for Corrosion Protection” has been launched as a means to present recent developments on any type of advanced coatings for corrosion protection. Fifteen contributions have been collected on metallic, inorganic, polymeric and nanoparticle enhanced coatings providing corrosion protection as well as partly other functionalities.

Corrosion is an important issue in many industrial fields. It leads to high economic losses of 3–4% of the GDP of an industrialized country year by year. Adequate corrosion protection is therefore essential in many applications. Among others, coatings are by far the most important technology for corrosion protection of metallic surfaces.

In the very traditional field of coatings for corrosion protection in the last years a deeper understanding of mechanisms of the protective action and corrosion mechanisms of and below protective coatings has been gained. This was necessary due to upcoming environmental and health issues for some well-established compounds used in former coating systems, e.g., lead or chromates, which have been banned from industrial application. This lead as well to a large amount of research in the field of advanced coating systems for corrosion protection.

This situation is the case for all the different types of protective coatings that are typically used. Novel metallic coatings, e.g., novel zinc alloys are under development, as well as novel pretreatment systems or passivating chemicals avoiding the use of chromates. The upcoming chemical nanotechnology fosters the development of hybrid or inorganic sol-gel coatings, as well as of nanoparticles and nanocapsules to be used as fillers in coating systems. This has also led, in recent years, to the development of novel self-healing and smart coatings. Furthermore, nowadays, bio-based substances are becoming increasingly used for organic coatings. Last but not least, new anodizing processes have also been developed in the frame of an increased use of light metals for light weight construction.

The special issue “Advanced Coatings for Corrosion Protection” has been proposed as a means to present recent developments on any of these types of advanced coatings for corrosion protection. Thus, 15 contributions have been collected on metallic, inorganic, polymeric and nanoparticle enhanced coatings providing corrosion protection as well as partly other functionalities.

Among all of them, inorganic coatings stand out for the number of contributions being submitted to this special issue; however, these are of many different types and for different applications. The thinnest but often quite effective type of an inorganic coating may be a passivating oxide layer. The most commonly used passivating agent to develop such an oxide layer is nitric acid. Lara-Banda et al. investigated an environmentally friendly alternative for the passivation of 15-5 and 17-5PH stainless steels based on citric acid [1]. It could be shown that, for both types of steel, the passive layer formed in citric acid as passivating solution had very similar characteristics to that formed with nitric acid.

Much thicker oxide layers can be obtained by anodizing techniques especially on light metals. Besides the conventional anodizing treatment at rather low voltages plasma-electrolytic oxidation (PEO) or micro arc oxidation (MAO), leading to ceramic oxide layers, has become an increasingly important alternative, being the topic of three contributions in this special issue [2,3,4]. Yao et al. prepared different MAO coatings on the magnesium alloy AZ91D [2]. It was found that especially a brown coating doped with Cu is able to significantly reduce the corrosion of magnesium parts in marine environments. Magnesium alloys to be used as biodegradable implant materials are the background of the paper by Anawati et al. [3]. Therefore, they investigated not only the corrosion resistance of PEO coatings, but also their ability to form bone mineral apatite. It was concluded that the alloying element Ca should be limited to 1 wt% as the excess tended to degrade the corrosion resistance and apatite-forming ability of the PEO coating. Kyziol et al. investigated the influence of MAO coatings on the stress corrosion cracking susceptibility of an AlMg6 alloy [4]. The pores in the MAO coating were insignificant and of limited depth. Therefore, the coating could increase the corrosion resistance.

Furthermore, inorganic coatings may be obtained from metal carbides [5], nitrides [6] or borides [7]. Jiang et al. used carburizing by a spark plasma sintering technique to enhance the erosion-corrosion resistance of tungsten in flowing coolant water [5]. W-Cr-C clad tungsten showed a different corrosion behavior than bare tungsten. Ti2AlN coatings were obtained by physical vapor deposition on ferritic steels and submitted to oxidation at a temperature of 700 °C by Gröner et al. [6]. The oxide scale of α-alumina was able to reduce the permeability for hydrogen significantly. Hu et al. obtained boride cermet coatings on carbon steel by a laser cladding process to improve the corrosion and wear resistance [7].

Polymeric coatings are widely used in corrosion protection and several contributions deal with this type of coatings as well [8,9,10,11,12]. Aging of the coating in terms of chain scission and phase separation may change the protective properties with time, as shown by Che et al. for polyurea coatings in marine atmosphere [8]. A wide variety of polymeric and hybrid systems can be chosen for protective coatings. Miela et al. showed how materials analysis and molecular dynamics simulation may help to identify the best performing coating system for erosion and corrosion protection of hydraulic water valves [9]. Polymeric coatings may also be used to add further functionalities to a barrier-type protective coating. As such, superhydrophobic properties were generated by Ou et al. on a zinc coating [10] and by Zhang et al. on the aluminum alloy AA5083 [11], providing water-repelling and long-term corrosion resistant surfaces. A very classical application of organic coatings is the insulation of buried pipelines. The paper by Hong et al. addresses this application, focusing on the additional cathodic protection design for a pre-insulated pipeline in a district heating system using computational simulation [12].

Finally, metallic coatings are widely used as noble barrier layers or as sacrificial layers providing cathodic protection to the substrate. One of the latter has been described by Tong et al. [13]. They produced a ZnAl diffusion layer on carbon steel by a mechanical energy aided diffusion method and characterized its corrosion behavior. On the other hand, barrier-type coatings may be reinforced by incorporation of nanoparticles into the coating matrix. Xu et al. demonstrated that the mechanical properties and the corrosion resistance of an aluminum foam can be improved by the electrodeposition of a NiMo coating that has been reinforced by SiC/TiN nanoparticles [14]. Furthermore, Fu et al. studied the effect of doping a NiFeCoP coating with cerium dioxide nanoparticles [15]. With an increased concentration of nano-CeO_2_ in the composite coating, its corrosion resistance increased as well.
